# Advanced statistical approaches in tuberculosis diagnosis and treatment outcomes in Africa: a systematic review

**DOI:** 10.1186/s41182-026-01020-7

**Published:** 2026-08-03

**Authors:** Olalekan John Okesanya, Tolutope Adebimpe Oso, Blessing Olawunmi Amisu, Yakub Burhan Abdullahi, Mohamed Mustaf Ahmed, Uthman Okikiola Adebayo, Yusuf Hared Abdi, Christian Joseph N. Ong, Olaniyi Abideen Adigun, Jerico Bautista Ogaya, Don Eliseo Lucero-Prisno

**Affiliations:** 1 Department of Medical Laboratory Science, Neuropsychiatric Hospital, Aro, Abeokuta, Ogun State Nigeria; 2 Department of Public Health and Maritime Transport, Faculty of Medicine, University of Thessaly, Volos, Greece; 3 Department of Medical Laboratory Science, McPherson University, Seriki Sotayo, Ogun State Nigeria; 4 Department of Medical Laboratory Science, IFPF Hospital, Maryland, Lagos, Nigeria; 5 Center for Health Research and Innovation, Somali National University, Mogadishu, Somalia; 6 Mogadishu Institute of Health, Mogadishu, Somalia; 7 Faculty of Health Sciences, Salaam University, Mogadishu, Somalia; 8 Faculty of Data Science, SIMAD University, Mogadishu, Somalia; 9 School of Global Health, Faculty of Medicine, Chulalongkorn University, Bangkok, Thailand; 10 Department of Medical Laboratory Services, Federal University of Health Sciences Teaching Hospital, Ila Orangun, Nigeria; 11 Department of Digital Health, Global Health Focus Africa, Kigali, Rwanda; 12 Department of Public Health, Faculty of Medicine and Health Sciences, Hormuud University, Mogadishu, Somalia; 13 Faculty of Medicine and Health Science, Hormuud University, Mogadishu, Somalia; 14 Department of Biology, College of Science, De La Salle University, Manila, Philippines; 15 Department of Medical Laboratory Science, Kwara State University, Malete, Kwara State Nigeria; 16 Department of Medical Technology, Institute of Health Sciences and Nursing, Far Eastern University, Manila, Philippines; 17 Center for University Research, University of Makati, Makati City, Philippines; 18 Department of Global Health and Development, Faculty of Public Health and Policy, London School of Hygiene and Tropical Medicine, London, UK; 19 Research Office, Palompon Institute of Technology, Palompon, Leyte Philippines

**Keywords:** Tuberculosis, TB, *Mycobacterium tuberculosis*, Africa, Bayesian models, Machine learning, Spatiotemporal analysis, Systematic review

## Abstract

**Background:**

Tuberculosis (TB) remains a major public health challenge in Africa; however, conventional statistical approaches often fail to capture diagnostic uncertainty, spatial heterogeneity, and complex disease dynamics, limiting evidence-based decision-making. This review synthesizes the application and performance of advanced statistical and computational methods for TB diagnosis and treatment outcomes in Africa.

**Methods:**

Following the PRISMA 2020 guidelines, we systematically searched PubMed and Scopus for peer-reviewed studies (2010–2025) conducted in Africa that applied advanced methods, including Bayesian models, machine learning (ML) algorithms, spatiotemporal analyses, time-series models, and multistate or survival frameworks. Study selection, data extraction, and quality appraisal were independently conducted by multiple reviewers using the Joanna Briggs Institute tools. Findings were synthesized narratively because of substantial methodological heterogeneity.

**Results:**

Twenty-seven studies from nine African countries were included. Bayesian hierarchical, geostatistical, and latent class models improved estimation of TB incidence, mortality, and diagnostic accuracy by accounting for uncertainty, imperfect reference standards, and sparse data. Spatiotemporal analyses consistently identified geographic TB and TB–HIV hotspots linked to low Bacillus Calmette–Guérin vaccine coverage, illiteracy, and urban crowding as risk factors. Time-series models quantified the significant decline in TB notifications during the COVID-19 disruptions. ML approaches, particularly random forests, outperformed traditional regression in predicting latent TB infection and treatment outcomes. Drug resistance, notably to bedaquiline and levofloxacin, showed clear spatial clustering and dynamic progression patterns.

**Conclusions:**

Advanced statistical and computational methods are increasingly improving TB diagnosis, prognosis, and treatment insights in Africa by capturing complex patterns beyond classical models, but their broader impact is limited by data gaps, heterogeneity, and insufficient validation, highlighting the need for high-quality, multi-country research and stronger implementation frameworks.

**PROSPERO ID:**

CRD420251160248

## Introduction

Tuberculosis (TB) remains a leading cause of infectious mortality, with an estimated 10.7 million people falling ill with TB in 2024 and 1.23 million deaths recorded globally, representing the first decline in incident cases since the COVID-19 pandemic [1]. Although global incidence has declined, progress is uneven, and the African region continues to bear a disproportionate burden despite achieving a 24% reduction in incidence since 2015 [2, 3]. Eight countries, including Nigeria and the Democratic Republic of Congo, accounted for two-thirds of global cases, highlighting persistent transmission hotspots in sub-Saharan Africa [3]. Control efforts are limited by weak surveillance systems that lead to significant underreporting, alongside a growing burden of multidrug-resistant TB (MDR-TB) and rifampicin-resistant TB (RR-TB). In 2021, an estimated 450,000 cases of RR-TB or MDR-TB were reported globally, reflecting the persistent challenge of drug-resistant forms of the disease [4, 5]. Limited laboratory capacity for culture and drug susceptibility testing further delays diagnosis and treatment monitoring, contributing to loss to follow-up [5]. These challenges create a complex, data-sparse environment in which advanced analytical approaches may offer insights that are beyond those of conventional regression models.

Recent methodological advances including Bayesian latent class models, integrated nested Laplace approximation (INLA), machine learning (ML) algorithms such as random forests and support vector machines, and spatiotemporal geostatistical frameworks have shown promise in addressing diagnostic uncertainty, heterogeneity, and prediction gaps [6]. The INLA has enabled robust survival modeling of TB–HIV co-infection, clarifying time-to-co-infection risks across diverse populations [7]. ML algorithms, such as decision trees, random forests, and support vector machines, have achieved over 90% accuracy in predicting treatment outcomes among drug-resistant TB cohorts [8]. Spatial Bayesian models have mapped clusters of TB–HIV co-infection, generating granular geographic intelligence for targeted interventions [9]. Time-series approaches, including autoregressive integrated moving average (ARIMA) and multistate Markov models, have been applied to forecast incidence trends and simulate intervention impacts; however, comprehensive syntheses of their use in African settings remain limited [10, 11].

Despite these advancements, most TB studies in Africa continue to rely on classical regression and descriptive statistics, restricting their ability to capture latent disease dynamics, diagnostic imperfections, and individualized risk profiles [12]. Evidence highlights gaps in the comparative assessments of Bayesian hierarchical models, latent class analyses, ML classifiers, and survival models, particularly regarding their relevance for decision-making in resource-limited environments [12]. Data challenges, such as missing variables, inconsistent reporting, and variable follow-up, also hinder the uptake of advanced methods [5]. Despite the urgency of achieving the World Health Organization (WHO) End TB Strategy targets, there is limited evidence on balancing model complexity with operational feasibility or on strategies for integrating these approaches into a national program [13]. Thus, this systematic review aims to evaluate the application, performance, and limitations of advanced statistical and computational methods in TB diagnosis, prognosis, and treatment outcomes across Africa, while identifying research gaps and comparing their use in different settings.

## Methodology

### Study design and reporting

This review was developed following the Preferred Reporting Items for Systematic Reviews and Meta-Analyses 2020 guidelines (S1 File). The protocol was registered in the International Prospective Register of Systematic Reviews under the registration number CRD420251160248.

### Information sources, search strategy and study selection

A comprehensive literature search was conducted using the PubMed and Scopus database to identify peer-reviewed studies applying advanced statistical or computational methods to TB diagnosis, prognosis, or treatment outcomes in African settings. To enhance the comprehensiveness of the search, the reference lists of all studies meeting the final inclusion criteria were manually screened to identify additional relevant publications that may not have been captured through the electronic search. The search strategy used the following Scopus query: TITLE-ABS-KEY ((“statistical model*” OR “Bayes*” OR “spatial model*” OR “time series” OR “survival analysis” OR “multistate model” OR “machine learning” OR “random forest” OR “latent class” OR “geostatistic*” OR “spatiotemporal*”) AND (“tuberculosis” OR “TB” OR “Mycobacterium tuberculosis”) AND (“Africa” OR “sub-Saharan” OR “Nigeria” OR “Ethiopia” OR “South Africa” OR “Kenya” OR “Ghana” OR “Tanzania” OR “Mozambique” OR “Uganda” OR “Zambia” OR “Zimbabwe” OR “Cameroon” OR “Senegal” OR “Malawi” OR “Congo” OR “Botswana” OR “Sudan” OR “Algeria” OR “Tunisia”)). The following PubMed search strategy was also applied**:**

((“statistical model*” OR “Bayes*” OR “Bayesian” OR “spatial model*” OR “time series” OR “survival analysis” OR “multistate model” OR “machine learning” OR “random forest” OR “latent class” OR “geostatistic*” OR “spatiotemporal*”)

**Fig. 1:**
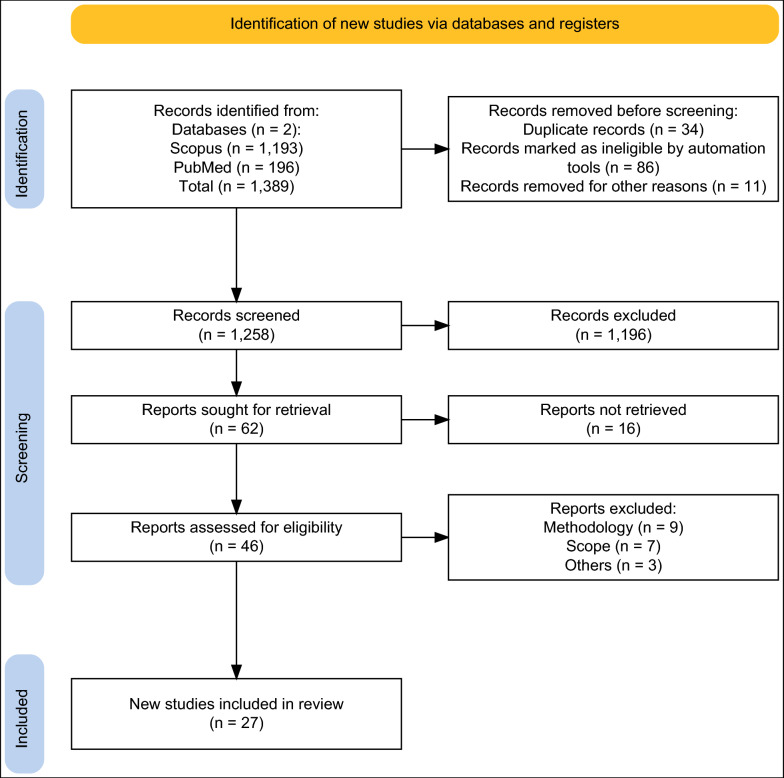
PRISMA flowchart of included studies

AND (“tuberculosis”[MeSH Terms] OR “tuberculosis”[Title/Abstract] OR “TB”[Title/Abstract] OR “Mycobacterium tuberculosis”[Title/Abstract]) AND

(“Africa”[MeSH Terms] OR “Africa”[Title/Abstract] OR “sub-Saharan Africa”[Title/Abstract] OR “Nigeria”[Title/Abstract] OR “Ethiopia”[Title/Abstract] OR “South Africa”[Title/Abstract] OR “Kenya”[Title/Abstract] OR “Ghana”[Title/Abstract] OR “Tanzania”[Title/Abstract] OR “Mozambique”[Title/Abstract] OR “Uganda”[Title/Abstract] OR “Zambia”[Title/Abstract] OR “Zimbabwe”[Title/Abstract] OR “Cameroon”[Title/Abstract] OR “Senegal”[Title/Abstract] OR “Malawi”[Title/Abstract] OR “Congo”[Title/Abstract] OR “Botswana”[Title/Abstract] OR “Sudan”[Title/Abstract] OR “Algeria”[Title/Abstract] OR “Tunisia”[Title/Abstract])). The search strategy focused on identifying studies based on statistical and computational methods rather than restricting by study design. Consequently, descriptive, retrospective, and prospective studies were included if they applied advanced statistical approaches relevant to TB outcomes. Truncation operators (e.g., *Bayes*, *geostatistical*, *spatiotemporal*) were used to maximize retrieval sensitivity. To further reduce the risk of missing country-specific studies that may not explicitly reference “Africa” in titles or abstracts, the geographic search strategy was expanded to include the names of individual African countries as well as major sub-regional identifiers (e.g., “sub-Saharan Africa”, “East Africa”, and “West Africa”). The study selection process is depicted below (Fig. 1).

### Eligibility criteria

Studies were selected based on predefined inclusion and exclusion criteria. We included peer-reviewed studies published in English between 2010 and 2025 that applied advanced statistical or computational methods to TB diagnosis, prognosis, or treatment outcomes in African settings. Although the search covered the period from 2010, all studies meeting inclusion criteria were published from 2018 onwards. Earlier studies (2010–2017) were excluded because they either did not apply advanced methods, focused on irrelevant TB outcomes, or did not meet other predefined eligibility criteria. Studies were included regardless of the population subgroup, provided they involved patients with TB or populations relevant to TB control. Where studies applied classical statistical methods alongside advanced approaches, these were included for descriptive comparison. However, formal comparison of study findings was not a primary objective of this review. The outcomes were categorized into three groups: diagnostic outcomes, which included studies evaluating diagnostic accuracy, screening performance, or test validation; prognostic or predictive outcomes, which encompassed studies assessing disease risk, incidence, forecasting, or prediction of future events; and treatment-related outcomes, which involved studies examining treatment response, recovery, mortality, drug resistance, or treatment success. The exclusion criteria included non–peer-reviewed publications, such as commentaries, editorials, opinion pieces, conference abstracts, and grey literature. Studies that did not apply advanced statistical or computational approaches in a TB-related context or were conducted outside Africa were excluded. Articles without full-text availability were also excluded.

### Data extraction

A standardized data extraction form was developed and pilot-tested to ensure clarity, completeness, and consistency among reviewers. Two reviewers (O.B.A., and J.B.O.) independently extracted data from all eligible studies using a pretested data extraction form, which captured key study characteristics, such as authorship, publication year, country of study, study design and population characteristics. The form also documented the specific advanced statistical or computational method applied, such as Bayesian modeling, ML algorithms, spatial analysis, time-series modeling, or multistate frameworks. Additional extracted elements included methodological performance metrics (e.g., diagnostic accuracy and predictive validity), key findings, and stated limitations. Studies were also assessed for contextual factors relevant to TB control in African settings, such as data quality challenges, computational feasibility and applicability to resource-limited environments. Any discrepancies in the extracted data across reviewers were resolved through discussion or, when necessary, by consultation with a third reviewer (O. J. O.) who adjudicated disagreements.

### Data synthesis

Given the substantial heterogeneity in study designs, populations, statistical methods, and outcome measures, a meta-analysis was not appropriate. Therefore, findings were synthesized narratively and thematically to allow meaningful integration of diverse methodological approaches and outcomes.

### Quality assessment

The methodological quality and risk of bias of all included studies were assessed independently by two reviewers (U.O.A and O.J.O) using the Joanna Briggs Institute (JBI) critical appraisal checklists appropriate for each study design with discrepancies resolved through consensus. These tools provide a structured framework for evaluating potential sources of bias across key domains, including participant selection, data collection, and outcome measurement. Using the JBI checklists ensured a transparent, systematic, and design-specific appraisal, allowing for a rigorous assessment of the study’s credibility and the reliability of the reported findings. The methodological quality of the included studies ranged from moderate to high. Most studies clearly defined inclusion criteria, used appropriate measurement methods, and applied suitable analytical techniques. Common limitations included incomplete reporting of confounding control, limited discussion of selection bias, and occasional ambiguity in participant recruitment. These methodological aspects were carefully considered during data synthesis and interpretation. Studies that adhered to a greater proportion of JBI criteria were given enhanced interpretative weight, whereas findings from studies with acknowledged limitations were contextualized accordingly.

## Results

### Overview of included studies

The 27 included studies covered 9 African countries which are South Africa, Ethiopia, Ghana, Mozambique, Tanzania, Zambia, Sudan, Algeria, and Botswana. Study designs varied widely, encompassing retrospective cohort designs, ecological and facility-based designs, modeling designs, time-series analyses, predictive modeling designs, diagnostic validation designs, and experimental designs (Table 1). Studies were categorized into modeling/time-series studies and comparative analysis studies based on the analytical approaches applied. Most included studies (*n* = 23) utilized advanced statistical and computational methods, including Bayesian hierarchical models, spatiotemporal modeling, time-series analyses, and ML approaches, to estimate TB incidence, forecast trends, identify high-risk geographic areas, and evaluate treatment outcomes. In contrast, a smaller number of studies (*n* = 4) applied classical statistical methods, primarily for descriptive comparison, risk estimation, or benchmarking purposes. Sample populations varied widely across studies, ranging from children under five years to adults aged 15 years and above [14, 15]. Some studies focused on specific populations, including HIV-positive individuals [7, 16], drug-resistant TB patients [17], and TB-suspected populations [18]. Population sizes also varied substantially, from small cohorts of 35 participants [19], to large national datasets exceeding 46,000 individuals [20, 21]. These variations highlight the diversity of study settings and populations included in this review.

**Table 1:** Summary of advanced statistical and conventional approaches to TB diagnosis and treatment outcomes in Africa

Citation	Country	Study design	Study classification	Population characteristics	Statistical/computational method	Outcome type	Key findings	Limitations
[16]	South Africa	Modeling	Modeling/time-series	–	Bayesian approach	Prognosis	The screening reduced the incidence of TB by 28.2%. 55% of TB cases and 69% of TB-related deaths were associated with HIV	The study concentrated on individuals aged 15 and above, restricting the general applicability for younger populations
[22]	Mozambique	Observational-retrospective	Modeling/time-series	–	Bayesian hierarchical Poisson regression models	Prognosis	Tuberculosis incidence varies significantly across Mozambique's districts	Ecological fallacy limits individual-level inference
[23]	Ethiopia	Modeling	Modeling/time-series	District-level data on HIV patients who attended healthcare facilities and received TB screening at their last visit	A Bayesian hierarchical modeling technique	Prognosis	National TB–HIV co-infection declined, though spatiotemporal analysis identified high-risk clusters	The data on HIV and TB–HIV co-infection case notifications obtained from the national HMIS may not accurately represent the true prevalence of these diseases in a specific district
[19]	Tanzania	Modeling	Modeling/time-series	Thirty-five samples were used	5-hypercubic model, with parameters estimated using the Baum–Welch algorithm	Treatment	Bedaquiline exhibits the greatest likelihood of acquiring drug resistance at 0.8660 with Levofloxacin following closely, with a probability of 0.134	The Tanzania study may have limited generalizability, as it used data from first-line treatment only
[14]	Ethiopia	Modeling	Modeling/time-series	The study examined children under 15 in rural Gedeo Zone, Ethiopia	Spatiotemporal random effects were incorporated into the model	Prognosis	Childhood tuberculosis incidence increased from 141 to 157 per 100,000 individuals	Limited adult TB data and misclassification in child TB cases hindered accurate analysis
[24]	Ghana	Modeling	Modeling/time-series	308 patients were registered, with deaths, defaults, transfers, lost to follow-up, and extended treatment cases treated as censored observations	Bayesian Continuous-Time Survival model was used for analysis while the Log-logistic model was selected as the best model	Treatment	Recovery rates were 79.32% for males and 87.32% for females. HIV/TB co-infection is linked to an 18.75% mortality rate	The main limitation is reliance on secondary data, which excludes key covariates like geo-spatial coordinates
[25]	Zambia	Time-series	Modeling/time-series	The study included individuals diagnosed with drug-susceptible TB	An interrupted time-series analysis was conducted using segmented linear regression	Prognosis	TB notifications declined by 22% after COVID-19 measures were implemented while HIV-positive individuals experienced a larger decline in notifications (− 36%)	Paper-based TB records and brief service interruptions may have caused data inaccuracies and underestimated TB impacts
[17]	Tanzania	Observational-retrospective cohort	Modeling/time-series	The study included TB patients resistant to first-line drugs, mostly aged 35–49 years	The study utilized Bayesian ZIP models for analysis	Treatment	TB drug resistance is geographically distributed with spatiotemporal dynamics	Reliability may be impacted by the small sample (652 drug-resistant patients)
[26]	Mozambique	Time-series	Modeling/time-series	–	A segmented time-series analysis using a negative binomial model was applied	Prognosis	TB notifications in Mozambique were heavily disrupted during COVID-19, with an estimated 17,147 cases (15.1%) potentially missed	Data gaps may have been exacerbated by COVID-19 measures, leading to an increase in TB cases that went unnoticed
[27]	Ghana	Modeling	Modeling/time-series	TB detection data sourced from the Ghana Health Service and National Tuberculosis Programme	Bayesian hierarchical models were utilized for analysis	Prognosis	TB infection risk was higher in the Northern zone and in Eastern and Greater Accra regions, with overall spatiotemporal risk concentrated in the Southern zone	Reliance on preexisting data may result in bias and input errors that compromise validity
[28]	Ethiopia	Observational- cross sectional	Comparative analysis	The study included 412 adults with TB symptoms, 60% of whom lived in rural areas	A negative binomial regression analysis was conducted	Prognosis	Twelve percent of patients missed TB diagnostics, primarily due to limited healthcare worker knowledge and patient awareness	A facility-based sample might not accurately represent the larger community
[29]	South Africa	Modeling	Comparative analysis	The study analyzed secondary data from four sources	Global and Local Moran's Indices methods and Spatial lag regression model were employed	Prognosis	TB death rates declined significantly from 179 to 60 per 100,000 population	The TB data source may have missed TB deaths due to the incompleteness of the Civil registration system
[21]	Ethiopia	Ecological	Modeling/time series	The population data include 46,697 adults aged 15 and above	A geostatistical model was applied using a Bayesian framework. Probability integral transform statistics were utilized for model assessment	Prognosis	The study identified spatial overlap of TB and undernutrition in Ethiopia	The study missed important covariates like economic factors, which limited findings
[30]	Sudan	Experimental	Modeling/time series	–	The study employed Seasonal Trend decomposition using LOESS (STL) and linear regression	Prognosis	Weekly data from Gedaref showed STL + regression outperformed ARIMA, reducing root mean square error by 81.9% from 2986.85 to 540.95	The ARIMA model underperforms due to its reliance solely on previously observed temporal patterns
[31]	South Africa	Modeling	Modeling/time series	Data from a rural Eastern Cape facility captured demographics, health status, and latent TB infection (LTBI) awareness and testing	Predictive modeling used logistic regression, with random forests applied to improve accuracy	Treatment	Random forests perform better in terms of accuracy and F1-score than logistic regression	Recall bias may exist in self-reported LTBI data
[32]	Botswana	Experimental	Modeling/time-series	4331 participants were enrolled in the TB study	Infection source probabilities were estimated using a Bayesian approach, with logistic regression applied for odds ratios	Prognosis	Host factors influence infectious disease transmission, aiding intervention development	Biased logistic regression with low sensitivity weakened effect estimates
[6]	South Africa	Diagnostic validation study	Modeling/time-series	Eligible participants of the study were aged 15 years and older	Bayesian latent class analysis (LCA) was applied, using probit regression for dependent binary data	Diagnosis	Sensitivity and specificity estimates were 38.9% and 88.9%, respectively	The accuracy of Xpert Ultra's 15.6 CFU/ml LOD may be limited by its potential to overlook low-load TB cases
[33]	South Africa	Diagnostic validation study	Modeling/time-series	The study included 9,869 participants aged ≥ 15, eligible for testing if they had TB symptoms or abnormal chest X-rays	Bayesian LCA was used to estimate Pulmonary TB prevalence	Diagnosis	Bayesian LCA alleviates reference standards and verification biases	Assuming unverified people are negative could result in erroneous estimates
[34]	South Africa	Observational-retrospective	Modeling/time-series	The study analyzed 97 HIV-TB co-infected young adults aged 15 to 34 years	A time-homogeneous Markov modeling approach was used for analysis	Treatment	TB co-infection increases the risk of viral rebounding on antiretroviral therapy (ART)	The multistate model was unable to account for the nonlinear effects of age, necessitating classification
[9]	Ethiopia	Modeling	Modeling/time-series	In 2018, 55.32% of TB patients were male. 62.91% of HIV care patients screened for TB were female	The global Moran’s index, Getis-Ord $G_{i}^{*}$ local statistic, and Bayesian spatial modeling techniques were applied to analyze the data	Prognosis	The result of the study shows that TB among people living with HIV (PLHIV) and HIV among TB patients' prevalence was geographically heterogeneous	The data were aggregated at the district level; therefore, the findings of this study cannot be representative of small administrative units of the country or Kebele or household or individual level
[35]	Ethiopia	Observational- facility based	Modeling/time-series	Population data represent populations seeking medical treatment in local facilities	A Bayesian hierarchical model approach was applied	Prognosis	TB risk remained nearly constant throughout the study period. Most high-risk districts for HIV were also high-risk for TB	Case notification data were used as a proxy, potentially missing untreated individuals
[18]	Ethiopia	Observational- facility based	Modeling/time-series	A total of 832 pulmonary TB-suspected patients participated in the study. 451 (54.2%) of the patients were male. 589 (70.8%) were rural residents	A retrospective Bernoulli model identified hotspot districts, and logistic regression analyzed patient characteristics and smear positivity	Prognosis	14.3% of presumptive Pulmonary TB patients were smear positive	The study was facility-based, which limits the ability to establish causal relationships between predictors and outcome variables
[7]	Ethiopia	Observational- retrospective cohort	Modeling/time series	The study included 421 HIV/AIDS patients, over half of whom were female, followed between January 2016 and December 2020	Bayesian parametric survival models were applied	Prognosis	About one-quarter of HIV/AIDS patients were co-infected with TB, with females comprising just over half of the co-infected cases	Important factors such as ART adherence status, ART treatment regimen, diabetes mellitus, viral load, and hypertension were not included in the study
[20]	Ethiopia	Ecological	Modeling/time-series	TB prevalence data came from Ethiopia’s 2010–2011 national survey of 46,697 adults and adolescents across all regions	The Bayesian geostatistical model was utilized for analysis. Binary logistic regression was used for parameter estimation	Prognosis	High TB prevalence in Ethiopia coincides with low BCG coverage, particularly in areas with poor access to health facilities, indicating gaps in vaccination services	The TB data covered fewer districts than the data used for BCG, which may affect the robustness of the findings
[15]	Mozambique	Ecological	Modeling/time-series	The study included all children under five newly diagnosed with TB from 2016 to 2020	The analysis used hierarchical Bayesian Poisson regression and spatial modeling to identify geographic patterns	Prognosis	TB incidence showed regional hotspots, increased with higher illiteracy, and was associated with greater healthcare facility density	Spatial bias may have resulted from the use of surveillance data, which may have underreported TB in underprivileged groups
[36]	Algeria	Observational-retrospective	Modeling/time series	–	Predictive modeling used Box-Jenkins methodology with SARIMA for time-series analysis and Moran’s I to assess spatial autocorrelation	Prognosis	From 1982 to 2019, smear-positive pulmonary TB notifications fell by 62.2%, while extrapulmonary TB notifications rose by 91.3%, with SPPTB hotspots in the northwest	Important TB clustering factors (socioeconomic, environmental, and climate) were not included because of data unavailability
[37]	Ethiopia	Modeling	Modeling/time-series	TB and HIV population data were sourced from the Health Management Information System and the web-based District Health Information System	Bayesian spatiotemporal modeling with a Poisson distribution was used to analyze TB cases	Prognosis	TB notifications (2018–2022) peaked early in the year, fell mid-year, and dropped sharply during COVID-19, totaling 206,278 cases	Centralized data were used, which might have underreported TB in some districts

*TB* tuberculosis, *HIV* human immunodeficiency virus, *MDR-TB* multidrug-resistant tuberculosis, *RR-TB* rifampicin-resistant tuberculosis, *ML* machine learning, *ARIMA* AutoRegressive Integrated Moving Average, *SARIMA* Seasonal AutoRegressive Integrated Moving Average, *STL* seasonal-trend decomposition using LOESS, *BCG* Bacillus Calmette–Guérin, *ART* antiretroviral therapy, *LCA* latent class analysis, *ZIP* zero-inflated Poisson, *HMIS* Health Management Information System, *LOD* limit of detection, *COVID-19* coronavirus disease 2019, *PLHIV* people living with HIV, *ART* antiretroviral therapy

### Statistical methods applied

A wide range of advanced statistical and computational methods were applied across the included studies. Bayesian-based approaches were the most frequently used, including Bayesian hierarchical models, Bayesian Poisson regression, Bayesian spatial modeling, and Bayesian survival models [7, 15, 22, 23, 27, 35, 37]. These methods were commonly applied to estimate TB incidence, identify geographic clusters, and evaluate treatment outcomes. Spatiotemporal and geostatistical approaches were also widely used, including hierarchical Bayesian spatial models, geostatistical modeling, Moran’s index, spatial lag regression, and Getis–Ord statistics [9, 20, 21, 29, 36]. These approaches were primarily employed to identify geographic clustering and spatial heterogeneity of TB incidence and mortality. Time-series approaches included segmented regression, interrupted time-series analysis, seasonal decomposition using Seasonal-Trend decomposition using Loess (STL), and Seasonal AutoRegressive Integrated Moving Average (SARIMA) modeling [25, 26, 30, 37]. These methods were commonly used to evaluate temporal trends and assess the impact of COVID-19 disruptions on TB notifications. Predictive modeling approaches included ML techniques such as random forests, logistic regression, and Baum–Welch algorithms [19, 31]. However, ML performance varied by dataset size and validation method, with some studies relying on small single-country datasets and internal validation only [19, 31]. Additionally, Markov models and zero-inflated Poisson models were used to model disease progression and drug resistance patterns [17, 34]. Diagnostic accuracy studies applied Bayesian latent class analysis to improve estimation of sensitivity and specificity in the absence of a gold standard [6, 33]. Finally, classical statistical approaches such as logistic regression, negative binomial regression, and parametric survival models were also applied, primarily for comparative analyses and risk estimation [28, 31, 32]. Although some of the cited methodological references are not TB-specific, they were included as they provide foundational statistical and computational frameworks (including Bayesian, ML, survival, and time-series methods) widely applied in TB epidemiological modeling and outcome prediction.

### Outcome types

The included studies addressed three primary outcome types: prognosis, diagnosis, and treatment outcomes. Several studies focused on prognostic outcomes, including TB incidence, mortality, geographic clustering, and temporal trends, often using Bayesian hierarchical models, spatial analyses, and time-series approaches [15, 21–23, 25, 36]. These studies identified high-risk populations, geographic hotspots, and temporal variations in TB burden. Diagnostic outcomes were evaluated in some studies using Bayesian latent class analysis and predictive modeling techniques, which improved diagnostic accuracy and addressed limitations associated with imperfect reference standards [6, 33]. These approaches enhanced sensitivity and specificity estimation, particularly in settings with limited laboratory capacity. Treatment-related outcomes included studies assessing treatment success, recovery rates, drug resistance, and disease progression using survival models, Markov models, and ML algorithms [19, 34]. These studies identified factors associated with treatment outcomes, including HIV co-infection, drug resistance, and demographic characteristics.

### Key findings

The included studies revealed several consistent patterns across geographic regions, populations, and methodological approaches. Bayesian hierarchical and spatial modeling studies identified significant geographic clustering of TB in Mozambique, Ethiopia, and Ghana, with high-risk areas often concentrated in districts with limited healthcare access, low literacy levels, and poor vaccination coverage [15, 20, 22, 27]. Similarly, spatial modeling in Ethiopia demonstrated overlap between TB incidence and undernutrition, highlighting socioeconomic determinants of disease burden [21]. Studies using spatial clustering methods, including Moran’s index and spatial lag regression, further confirmed non-random geographic patterns in TB mortality and incidence [9, 29, 37]. In Zambia, TB notifications declined by 22% following COVID-19 measures, with larger declines observed among HIV-positive individuals [25]. Similarly, Mozambique reported approximately 17,147 missed TB cases during pandemic disruptions [26]. Seasonal time-series modeling also demonstrated substantial declines in TB detection during COVID-19 periods across some settings [30, 36]. Findings reported strong associations between HIV co-infection and TB outcomes. In South Africa, over half of TB cases and TB-related deaths were associated with HIV [16]. Bayesian survival modeling in Ethiopia also reported that approximately one-quarter of HIV/AIDS patients were co-infected with TB, with increased mortality risk [7]. Similarly, survival analysis in Ghana demonstrated higher mortality among TB/HIV co-infected individuals [24]. Advanced modeling studies identified patterns in drug resistance and treatment outcomes. A Baum–Welch model in Tanzania reported high probabilities of resistance emergence for bedaquiline and levofloxacin [19]. Spatial modeling also identified geographic clustering of drug-resistant TB cases [17]. A Markov modeling study demonstrated increased risk of viral rebound among HIV-TB co-infected individuals receiving treatment [34]. A predictive modeling study demonstrated improved performance of advanced computational approaches. Random forest models outperformed logistic regression for latent TB infection prediction in South Africa [31]. Bayesian latent class analyses improved diagnostic accuracy and reduced verification bias in diagnostic validation studies [6, 33]. Additionally, hybrid time-series modeling combining STL with regression substantially reduced prediction errors compared with ARIMA models [30]. Some studies identified sociodemographic factors influencing TB burden and treatment outcomes. Age, sex, HIV status, undernutrition, literacy levels, and access to healthcare services were consistently associated with TB incidence and mortality [15, 34]. A facility-based observational study also reported missed diagnostic opportunities linked to healthcare worker knowledge and patient awareness [28]. These findings demonstrate that advanced statistical and computational methods provide valuable insights into TB epidemiology, enabling improved prediction, diagnosis, and treatment outcome assessment across diverse African settings.

## Discussion

This systematic review synthesized evidence highlighting the application, performance, and limitations of advanced statistical and computational methods for TB diagnosis, prognosis, and treatment outcomes. The findings demonstrate that Bayesian hierarchical models, ML algorithms, and spatiotemporal analyses consistently provided more accurate and contextually relevant insights than classical methods. The TB burden in Africa shows marked geographic and demographic variability, with COVID-19 disruptions reducing diagnoses, elevated risk among HIV-co-infected and undernourished individuals, and notable patterns of drug resistance. Advanced methods, including Bayesian models, random forests, and STL regression, improved prediction, diagnosis, and understanding of treatment outcomes across diverse settings.

Globally, the COVID-19 pandemic led to a substantial decline in TB diagnoses due to disruptions in essential services, effectively reversing years of progress [38]. While sub-Saharan Africa largely returned to pre-pandemic notification levels by 2021, similar disruptions in Brazil, Spain, Italy, and China delayed diagnosis and increased disease severity [39–41]. COVID-19 also disrupted HIV/TB co-infection management, highlighting the need for resilient health systems during overlapping infectious disease crises [37]. Geographic heterogeneity in TB incidence and mortality is influenced by population density, urbanization, socioeconomic status, and healthcare access [42–44]. Africa, hotspots often coincide with poor Bacillus Calmette–Guérin (BCG) coverage and low literacy, whereas Asia and Latin America benefit from more comprehensive surveillance supporting finer spatial modeling [42, 43]. Undernutrition and HIV co-infection are major drivers of elevated TB mortality, compounded by limited access to healthcare [42]. Advanced spatial and temporal analyses in China and Thailand have revealed significant regional clustering, demonstrating the value of targeted interventions, although persistent data gaps limit similar applications in African settings [42, 45].

The convergence of advanced statistical and computational methods allows for a coherent explanation of TB patterns, while demonstrating superiority over traditional analytical techniques. Bayesian hierarchical and latent class models outperform conventional methods by explicitly accounting for uncertainty and measurement error, particularly in contexts with incomplete bacteriological confirmation, variable diagnostic sensitivity, and sparse surveillance data [6, 31, 46]. These methods yielded stable estimates of TB incidence, mortality, and drug resistance, revealing high-risk clusters masked by single-level analyses [6, 31, 46]. Spatiotemporal and geostatistical models further elucidate how structural determinants, such as urban crowding, low BCG coverage, poverty, and illiteracy, drive localized TB hotspots, while Moran’s Index and related spatial statistics confirm non-random clustering of both drug-susceptible and drug-resistant TB [47]. Time-series approaches, including segmented regression, SARIMA, and STL-based hybrid models, robustly quantified COVID-19’s disruptive effects on TB notifications and service utilization, outperforming simple pre–post comparisons [48].

Compared with classical statistical approaches such as logistic regression and Cox proportional hazards models, Bayesian methods demonstrated superior flexibility in handling sparse data, missing values, and diagnostic uncertainty, particularly in settings with limited laboratory confirmation. However, they are computationally intensive and require strong prior specification, which may limit their routine use in low-resource settings [6, 33]. Classical statistical methods remain valuable for hypothesis testing, interpretability, and ease of implementation, especially in routine public health settings. However, their limited capacity to model spatial heterogeneity, temporal dynamics, and latent structures reduces their effectiveness in complex TB epidemiological contexts [49, 50].

Regression-based survival and risk models, particularly Cox proportional hazards and multivariable logistic regression, elucidated links between undernutrition, HIV co-infection, delayed diagnosis, and elevated mortality by adjusting for confounding factors and modeling time-to-event outcomes [51]. ML methods, including random forests and hidden Markov-based Baum–Welch algorithms, captured complex, nonlinear interactions among host, environmental, and clinical variables, outperforming traditional regression in predicting latent TB infection, treatment outcomes, and resistance acquisition, especially in heterogeneous African populations. However, it is important to note that many reported high accuracy values were derived from internal validation on limited datasets, which may overestimate real-world predictive performance [52, 53]. Markov and zero-inflated Poisson models strengthened findings on drug resistance, handling transition probabilities and excess zeros, reflecting real-world treatment pathways [54, 55]. For example, bedaquiline resistance emerged primarily during treatment in South Africa, with cross-resistance complicating regimens [55, 56]. Spatial clustering of drug-resistant TB is evident in Africa, though limited laboratory capacity and fragmented surveillance restrict detailed modeling compared to Asia and Latin America [54, 55]. Despite these challenges, advanced computational and statistical methods provide accurate, unbiased, and policy-relevant evidence, improving diagnostic accuracy, risk stratification, targeted interventions, and ultimately TB control and treatment outcomes [42, 57].

### Implications for policy, practice and future research

The consistent identification of TB and TB–HIV hotspots in districts characterized by low BCG coverage, high illiteracy rates, and limited healthcare access in Ethiopia, Mozambique, Ghana, and South Africa highlights the importance of geographically targeted interventions, including strengthened primary healthcare outreach and closer integration of TB–HIV services in high-risk settings. Additionally, the increasing application of Bayesian hierarchical, spatiotemporal, multistate, and ML approaches in African TB research reflects growing regional analytical capacity and supports the integration of advanced methods into national TB control programs. These approaches could enhance real-time hotspot detection, resource allocation, targeted screening, and treatment monitoring [12, 35, 58]. Nevertheless, implementation in routine surveillance systems remains constrained by limited technical expertise, computational demands, and weaknesses in health data infrastructure, particularly in low-resource settings [12, 59]. Consequently, classical statistical methods continue to play an important complementary role in programmatic decision-making. Overall, the findings of this review align with the WHO End TB Strategy, particularly Pillars 2 and 3, by demonstrating how advanced analytical approaches can strengthen evidence-based surveillance, targeted interventions, research innovation, and TB control decision-making across African settings [60, 61].

### Limitations of the review

One study relied on secondary data sources, such as surveillance registries and facility-based records, increasing the risk of missing or inaccurate case identification [29]. Issues of sampling and generalizability were also common [18], there was also focus on narrowly defined populations, thereby limiting the broader applicability of the findings [19]. In addition, studies employing district-level or aggregated spatial data are susceptible to ecological fallacy, restricting inference at the individual level [9, 22]. Measurement and reporting errors, including gaps in TB notification, reliance on paper-based records, misclassification, and recall bias, further undermine data reliability [7, 25, 31]. Some  studies lacked key covariates such as ART adherence, socioeconomic status, environmental exposures, and comorbidities, which constrained comprehensive risk assessment and model robustness [7, 21, 36]. Additionally, the exclusion of grey literature, while consistent with our predefined eligibility criteria aimed at peer-reviewed evidence, may have resulted in the omission of relevant programmatic data and unpublished findings that could have enriched the synthesis. Finally, many included studies lacked external validation, as most models relied on single-country datasets and internal validation only. This limits their generalizability and transferability across diverse African settings [9, 15, 16, 19, 20, 27, 31, 37].

## Conclusion

This systematic review of 27 studies shows that advanced statistical and computational methods particularly Bayesian models, spatiotemporal analyses, time-series approaches, and multistate or survival frameworks are increasingly applied to TB diagnosis, prognosis, and treatment outcomes in the region. Across the included studies, the methods consistently provided richer inference than classical regression alone, by accounting for spatial clustering, imperfect reference standards, excess zeros, and complex time‑to‑event processes. Evidence suggests that Bayesian and ML approaches can enhance the estimation of diagnostic accuracy, measure service disruptions such as those caused by COVID-19, and identify high-risk geographic areas and patient subgroups that may benefit from targeted interventions. In addition, advanced survival and Markov models can help clarify patterns of treatment response and the emergence of drug resistance. At the same time, the field remains constrained by heterogeneous study designs, incomplete covariate data, limited external validation, and a concentration of research in a subset of African countries, which together restrict the generalizability and operational readiness of many models. Taken together, these findings support cautiously expanding the use of advanced statistical and computational tools within African TB programs, provided that they are implemented alongside investments in data quality, geocoded routine surveillance, and capacity‑building for analysts and decision‑makers. Future research should prioritize prospective, multi-country studies that integrate high-quality clinical, laboratory, and social data, while rigorously comparing advanced methods with simpler alternatives. Emphasis should also be placed on transparent reporting and robust validation to ensure that more complex models provide meaningful added value for TB control across diverse African settings.

## Data Availability

No datasets were generated or analyzed during the current study.
